# Consumption of a 12.5% carbohydrate after 8 h of fasting does not delay gastric emptying in healthy volunteers: a single-blind, randomized, non-inferiority trial

**DOI:** 10.3389/fnut.2026.1751422

**Published:** 2026-06-01

**Authors:** Gang Zhang, Xiaoyan Huang, Ji Feng, Wenchao Yin, Fu Yao

**Affiliations:** 1 Department of Anesthesia, Sichuan Provincial Orthopedic Hospital (Chengdu Sports Hospital and Chengdu Research Institute for Sports Injury), Chengdu, China; 2 Department of Operation Room, The Third People's Hospital of Chengdu, Chengdu, China

**Keywords:** cross-sectional area, ERAS, gastric volume, insulin resistance, ultrasound

## Abstract

**Objective:**

To evaluate the gastric safety, metabolic effects, and subjective comfort of consuming a 12.5% carbohydrate beverage 2 h before surgery in healthy volunteers after prolonged fasting, using point-of-care gastric ultrasound.

**Methods:**

This single-center, randomized, controlled, single-blind, non-inferiority trial assigned 66 healthy volunteers 1:1 to an experimental group (≤400 mL of the beverage after 8 h of fasting) or a control group (continued fasting). The primary outcome was the antral cross-sectional area (CSA) in the right lateral decubitus position, with a non-inferiority margin of 2.0 cm^2^. Secondary outcomes included gastric volume (GV), incidence of a “high-risk” stomach, blood glucose, insulin, homeostasis model assessment of insulin resistance (HOMA-IR), and visual analogue scale (VAS) scores for hunger and thirst.

**Results:**

The antral CSA in the experimental group was non-inferior to the control group at 2 h (T2) and 3 h (T3) post-ingestion (both *p* > 0.05). The incidence of a high-risk stomach showed no significant between-group difference at these times (both *p* = 0.500). The experimental group showed transient physiological elevations in blood glucose, insulin, and HOMA-IR at 1 h (T1) and T2 (all *p* < 0.001), which returned to levels comparable with the control group by T3 (all *p* > 0.05). Hunger and thirst VAS scores were significantly lower in the experimental group at T3 (*p* = 0.012).

**Conclusion:**

In healthy volunteers, preoperative carbohydrate administration appears relatively safe and effective in this population and may serve as a potential alternative to prolonged fasting. It does not delay gastric emptying, and effectively relieves hunger and thirst, supporting its potential inclusion in ERAS protocols for healthy individuals.

**Clinical trial registration:**

http://www.chictr.org.cn, identifier ChiCTR2100043166.

## 1 Introduction

Pulmonary aspiration is a rare but devastating complication during general anesthesia, which can directly lead to aspiration pneumonia, acute respiratory distress syndrome, multiple organ dysfunction, and even death, representing one of the leading causes of anesthesia-related mortality. Studies have reported that the incidence of pulmonary aspiration during anesthesia ranges from 0.1 to 19%. A prospective multicenter observational study covering 261 hospitals in Europe showed that the overall incidence of severe pulmonary aspiration during pediatric anesthesia was 0.1% (1), and aspiration-related perioperative deaths accounted for 9% of total anesthesia-related mortality (2, 3). After administration of muscle relaxants in general anesthesia, the lower esophageal sphincter relaxes and airway protective reflexes are abolished, making gastric contents prone to regurgitation and aspiration into the respiratory tract during spontaneous breathing or positive-pressure ventilation, resulting in severe pulmonary complications. Due to the lack of specific rescue strategies in clinical practice, prevention of perioperative pulmonary aspiration remains a core component of anesthetic management.

For a long time, strict preoperative fasting has been regarded as the primary measure to prevent pulmonary aspiration. However, accumulating evidence indicates that prolonged fasting induces pronounced thirst, hunger, anxiety, irritability, dehydration, and exaggerated stress responses, which further lead to endocrine disorders and increased insulin resistance. These changes ultimately impair perioperative homeostasis, delay postoperative recovery, and increase the risk of complications (4, 5). Insulin resistance disrupts metabolic homeostasis, causes negative nitrogen balance, weakens anti-infective capacity, and the associated hyperglycemia is closely linked to postoperative infections and adverse cardiovascular events. Studies have confirmed that preoperative oral carbohydrate administration attenuates postoperative stress responses and partially improves insulin resistance in patients undergoing major abdominal surgery.

To balance preoperative safety and patient comfort, the 2017 guidelines from the American Society of Anesthesiologists (ASA) on preoperative fasting explicitly recommend clear fluid intake up to 2 h before surgery. Relevant Chinese guidelines also suggest oral administration of 200–400 mL of carbohydrate beverage 2 h preoperatively. Preoperative carbohydrate intake 2 h before surgery has become a key component of Enhanced Recovery After Surgery (ERAS) protocols. Numerous studies have verified that this practice improves patient comfort, reduces stress levels, ameliorates insulin resistance, and accelerates postoperative recovery (6, 7).

Despite clear guideline recommendations, a considerable gap remains in clinical implementation. Multiple studies worldwide have shown that prolonged preoperative fasting is still prevalent. The median duration of solid fasting exceeds 13 h in Europe, 12–13 h in Japan, and 14–16 h on average in Chinese patients, all significantly longer than recommended by guidelines (8, 9). The main reasons include the widespread belief among clinicians and patients that “longer fasting is safer,” together with practical constraints such as uncertain surgical schedules and inefficient workflow management. However, controversy persists regarding whether oral carbohydrate intake 2 h preoperatively is truly safe and does not increase gastric volume or the risk of regurgitation and aspiration. Some studies have suggested that residual gastric volume may still be present even with fluid ingestion 2 h preoperatively (10), leaving clinicians uncertain about safety.

In recent years, point-of-care gastric ultrasound has emerged as a reliable, noninvasive, real-time, and reproducible tool for evaluating gastric contents, antral cross-sectional area (CSA), and aspiration risk. Its performance is comparable to radionuclide imaging, the gold standard for measuring gastric emptying, allowing objective assessment of gastric emptying status (11, 12). To date, high-quality randomized controlled trials are still lacking that strictly simulate clinical prolonged fasting in healthy individuals and use gastric ultrasound to evaluate the safety of preoperative carbohydrate administration 2 h before surgery.

Therefore, the present study enrolled healthy volunteers and used point-of-care gastric ultrasound to measure antral CSA and gastric volume. Using a non-inferiority design, we aimed to determine whether oral carbohydrate intake 2 h preoperatively is non-inferior to conventional prolonged fasting. We also evaluated its effects on insulin resistance and subjective symptoms such as hunger and thirst, to provide objective and reliable safety evidence for the clinical application of preoperative oral carbohydrate administration 2 h before surgery.

## 2 Methods

### 2.1 Study design

This was a single-center, randomized, controlled, single-blind, non-inferiority clinical trial conducted in healthy volunteers, aiming to evaluate the gastric safety of oral administration of 12.5% carbohydrate beverage 2 h after prolonged preoperative fasting. The study protocol was approved by the Medical Ethics Committee of Sichuan Provincial Orthopedic Hospital, and written informed consent was obtained from all participants. The trial was registered in the Chinese Clinical Trial Registry (ChiCTR2100043166) before implementation.

In the originally registered protocol of this study, the experimental group adopted a fixed-dose design with three groups (200 mL, 300 mL, and 400 mL), and the primary outcome indicator was the incidence of high-risk stomach. After ethical review and clinical feasibility assessment, the protocol was optimized to a two-group control design, where the experimental group adopted individualized on-demand intake (≤400 mL), which is consistent with clinical practice, ethical requirements and personalized medical principles.

Regarding the change of primary outcome indicator: there is no unified definition of “high-risk stomach,” which is highly controversial, with poor objectivity and repeatability. The cross-sectional area (CSA) in the right lateral decubitus position, directly measured by ultrasound, is a quantitative indicator with stability and strong repeatability. It is more in line with the purpose of this study to evaluate the safety of gastric emptying of preoperative carbohydrate drinks. Therefore, the primary outcome indicator was changed to CSA. Meanwhile, the incidence of high-risk stomach was set as an important secondary indicator for observation and discussion.2. Study Population.

#### 2.1.1 Inclusion criteria

- Age 18–60 years
- American Society of Anesthesiologists (ASA) physical status class I
- Body mass index (BMI) 18–28 kg/m^2^
- Good general health, free of metabolic or digestive diseases
- Able to cooperate with gastric ultrasound examination and blood sampling

#### 2.1.2 Exclusion criteria

- Pregnancy
- Diabetes mellitus or other endocrine/metabolic diseases
- Renal diseases
- Organic gastrointestinal diseases
- Use of medications affecting gastric motility or gastric acid secretion within the past 1 week
- Inability to tolerate fasting or ultrasound examination

### 2.2 Interventions

All participants followed a standard preoperative fasting protocol: after at least 8 h of fasting from solids and liquids, they entered the experimental procedure.

Eligible volunteers were randomly assigned at a 1:1 ratio using a computer-generated random number list:

- Experimental group (Group E, *n* = 33): After 8 h of fasting, participants orally ingested a 12.5% carbohydrate beverage (Kelun, China) *ad libitum* within a safe upper limit of 400 mL.
- Control group (Group C, *n* = 33): Participants continued fasting from solids and liquids.

### 2.3 Definition of time points

- T0: Immediately before carbohydrate ingestion in the experimental group / after 8 h of continuous fasting in the control group
- T1: 1 h after beverage ingestion in the experimental group / after 9 h of fasting in the control group
- T2: 2 h after beverage ingestion in the experimental group / after 10 h of fasting in the control group (primary time point)
- T3: 3 h after beverage ingestion in the experimental group / after 11 h of fasting in the control group

### 2.4 Gastric ultrasound assessment

All ultrasound examinations were performed in a single-blind manner by an anesthesiologist with more than 3 years of experience in point-of-care gastric ultrasound, who was unaware of group allocation throughout the study to minimize measurement bias.

**Figure 1:**
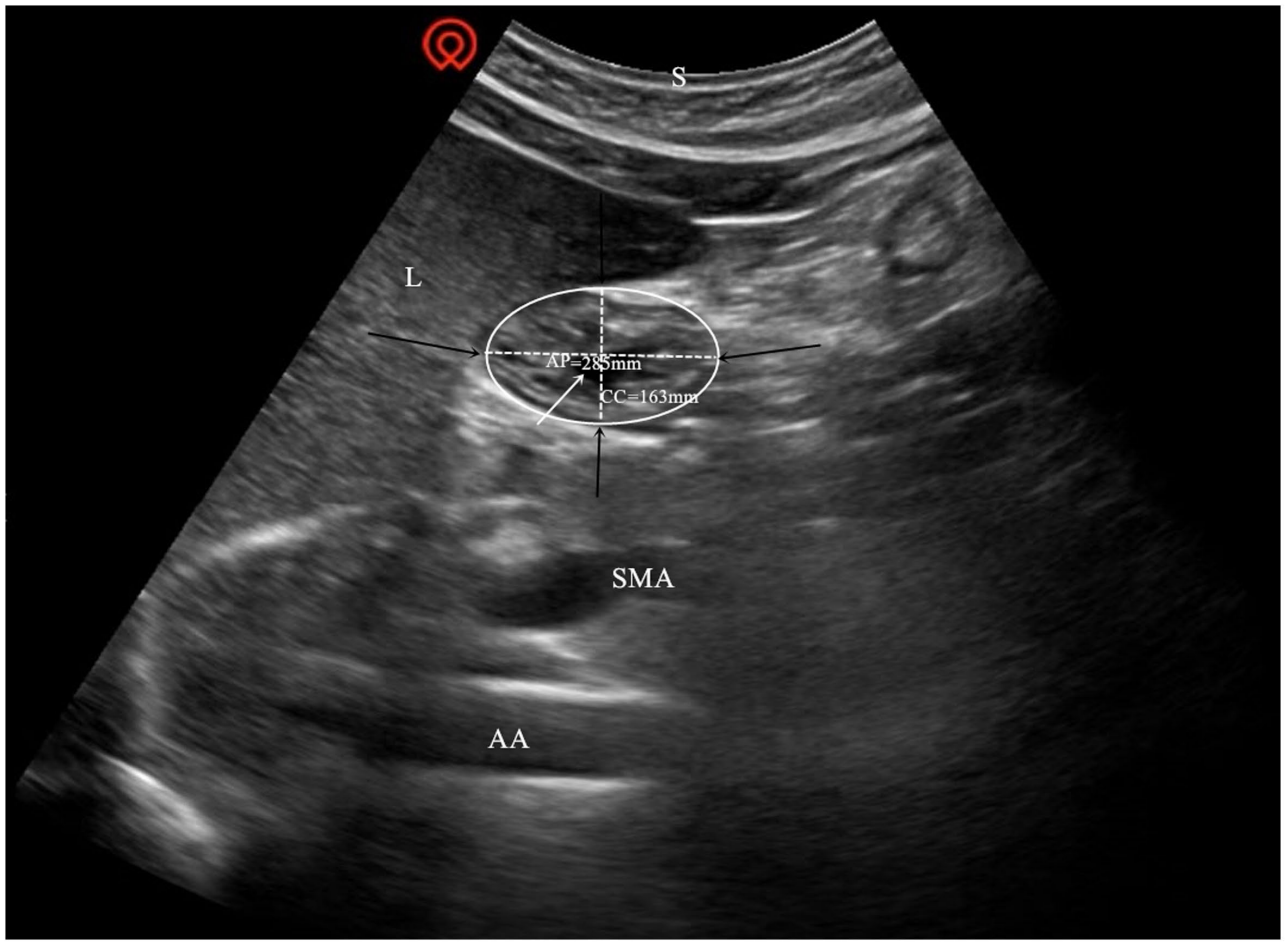
Ultrasound scan of the gastric antrum in the right decubitus position in a subject who was classified as Perlas 1. AP, antero posterior diameter; CC, craniocaudal diameter; S, skin; L, liver; SMA, superior mesenteric artery; AA, abdominal aorta. AP = 285 mm, CC = 163 mm.

A Navi U ultrasound system (Wisonic Inc., China) equipped with a low-frequency convex probe (2–5 MHz) was used. Standard views were obtained in the supine and right lateral decubitus positions, demonstrating the left lobe of the liver, superior mesenteric artery, and abdominal aorta.(Figure 1).

#### 2.4.1 Perlas semi-quantitative grading

- Grade 0: No gastric contents visible in either the supine or right lateral decubitus position
- Grade 1: No gastric contents in the supine position, but visible in the right lateral decubitus position
- Grade 2: Gastric contents visible in both positions

#### 2.4.2 Measurement of antral cross-sectional area (CSA)

In the right lateral decubitus position at end-expiration, the anteroposterior (AP) diameter and craniocaudal (CC) diameter of the antrum were measured three times each, and the mean values were used. CSA was calculated using the elliptical formula:


CSA(cm2)=π×AP(cm)×CC(cm)/4

#### 2.4.3 calculation of gastric volume (GV)

GV was estimated using the following formula:


$$\begin{array}{c} \mathrm{GV}\;(\mathrm{ml})=27.0+14.6\times \text{right lateral decubitus}\;\mathrm{CSA}\;(\mathrm{cm2})\\ -1.28\times \mathrm{age}\;(\text{years}) \end{array}$$

In accordance with international standards, calculated values ≤ 0 mL were corrected to 0 mL, representing an empty stomach (13, 14).

#### 2.4.4 Definition of high-risk stomach

A high-risk stomach was defined as meeting any one of the following criteria (11):

1. Perlas grade 2
2. Perlas grade 1 with CSA > 340 mm^2^
3. GV > 1.5 mL/kg

### 2.5 Primary and secondary outcomes

#### 2.5.1 Primary outcome

Antral CSA in the right lateral decubitus position (a continuous quantitative variable that is objective, stable, and highly reproducible).

#### 2.5.2 Secondary outcomes

1. Incidence of high-risk stomach
2. Blood glucose (BG) and insulin (INS) levels
3. Homeostasis model assessment of insulin resistance (HOMA-IR) = fasting BG (mmol/L) × fasting INS (mIU/L) / 22.5
4. Gastric volume (GV)
5. Visual analogue scale (VAS) scores for hunger, thirst, and anxiety (0–3 points)

### 2.6 Non-inferiority design and margin setting

This non-inferiority trial was designed to confirm that gastric emptying safety in the experimental group is not inferior to that in the traditional fasting group.

Based on the study by Perlas et al.: CSA of empty stomach (grade 0) was 3.6 ± 1.0 cm^2^, and CSA of low-risk stomach (grade 1) was 5.6 ± 1.4 cm^2^ (15). A non-inferiority margin of 2.0 cm^2^ was set (mean grade 1 CSA − mean grade 0 CSA = 5.6–3.6).

### 2.7 Sample size calculation

Based on pilot study data:

- Experimental group CSA at T2: 4.46 ± 1.51 cm^2^
- Control group CSA at T2: 3.56 ± 1.04 cm^2^
- Non-inferiority margin (NIM): 2.0 cm^2^
- Type I error rate *α* = 0.025 (one-sided)
- Power 1 − *β* = 0.90
- Using PASS 15 software, a total sample size of 60 participants was required. Considering a 10% dropout rate, 33 subjects were allocated to each group, resulting in a total sample size of 66, which is comparable to similar studies (16).

### 2.8 Randomization and blinding

Participants were randomly assigned at a 1:1 ratio using a computer random number generator, with allocation results stored in sealed opaque envelopes. Statisticians and ultrasound examiners were blinded to group allocation throughout the study; they only performed measurements and statistical analyses without involvement in intervention administration.

### 2.9 Statistical analysis

All statistical analyses were performed using SPSS software.

- Normally distributed continuous data were presented as mean ± standard deviation (SD). Between-group comparisons were performed using independent-samples *t*-test, with mean difference (MD) and 95% confidence interval (CI) reported.
- Non-normally distributed continuous data were presented as median (interquartile range), and between-group comparisons were performed using the Mann–Whitney *U* test.
- Categorical data were presented as counts/proportions. Between-group comparisons were performed using the chi-square test or Fisher’s exact test, with odds ratio (OR) and 95% CI reported.

Repeated-measures analysis of variance (RM-ANOVA) was used for repeated measurements including CSA, GV, BG, INS, and HOMA-IR. If the Mauchly test of sphericity was violated (*p* < 0.05), the Greenhouse–Geisser correction was applied. When a significant group × time interaction was detected, simple-effect analyses were performed with Bonferroni correction for multiple comparisons. All tests were two-sided, and *p* < 0.05 was considered statistically significant.

## 3 Results

### 3.1 Baseline characteristics of participants

**Figure 2:**
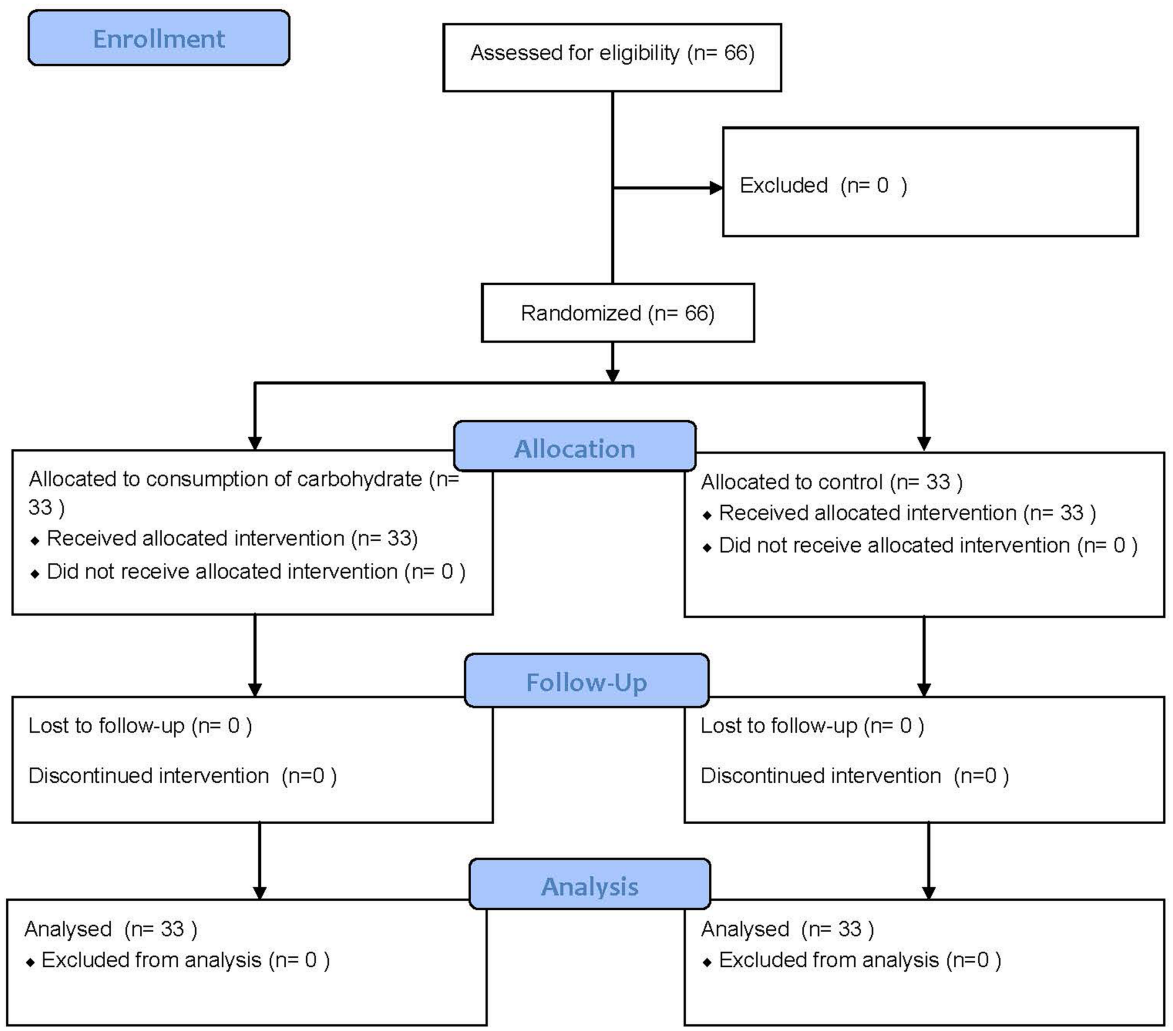
CONSORT flow diagram.

A total of 66 healthy volunteers were enrolled in this study, with 33 participants allocated to the experimental group and 33 to the control group. Since the primary outcome measure was changed to CSA of the gastric antrum, a recalculation of the sample size indicated a requirement of 66 participants. Therefore, 6 additional participants were recruited beyond the original 60, with their enrollment, interventions, and data collection procedures being entirely consistent with the previous participants. No participant dropped out during the entire study period (Figure 2). There were no statistically significant between-group differences in baseline characteristics, including height, weight, age, body mass index (BMI), and sex distribution (all *p* > 0.05), indicating well-balanced and comparable baselines between the two groups (Table 1).

**Table 1:** Comparison of subjects’ general data.

Baseline variable	Group E (*n* = 33)	Group C (*n* = 33)	MD (E-C) or OR (E/C)	95% CI of MD or OR		*P*
				LL	UL	
Height (cm)	170.27±9.29	170.15 ± 9.42	0.12	−4.48	4.72	0.958
Weight (Kg)	66.48 ± 12.57	70.58 ± 9.32	−4.09	−9.53	1.35	0.138
Age (years)	37.97 ± 11.06	38.00 ± 12.33	−0.30	−5.79	5.73	0.992
BMI (Kg/m^2^)	22.92 ± 3.76	24.54 ± 3.89	−1.61	−3.49	0.26	0.091
Sex(Male/Female, *n*)	19/14	15/18	1.27	0.79	2.04	0.460
Intake (ml)	221.23 ± 51.07					

MD, Mean difference; CI, Confidence interval; LL, Lower limit; UL, Upper limit; OR, Odds ratio.

### 3.2 Primary outcome

Antral Cross-Sectional Area (CSA) in the Right Lateral Decubitus Position (Table 2).

**Table 2:** Comparison of the outcomes.

Outcomes	Group E (*n* = 33)	Group C (*n* = 33)	MD (E-C) or OR (E/C)	95% CI of MD or OR		*P*
				LL	UL	
CSA (cm^2^)	P_Mauchly_ < 0.001, Fgroup*time=258.11, Pgroup*time<0.001					
T0	11.46±1.82	3.70±0.60	7.78	7.40	8.11	<0.001
T1	6.28±1.52	3.68±0.74	2.60	2.16	3.03	<0.001
T2	4.15±1.59	3.68±0.81	0.47	−0.15	1.10	0.137
T3	3.66±0.85	3.68±0.73	−0.13	−0.36	0.336	0.940
GV (ml)	P_Mauchly_ <0.001, Fgroup*time=260.49, Pgroup*time<0.001					
T0	145.70±14.72	32.46±17.39	113.24	105.31	121.16	<0.001
T1	70.08±19.22	32.16±18.13	37.91	28.72	47.10	<0.001
T2	39.09±20.21	32.33±21.49	6.76	−5.58	19.09	0.278
T3	31.09±19.43	32.09±18.72	−0.16	−9.12	9.43	0.973
Incidence of HRS (N)						
T0	33	4	8.25	3.29	20.67	<0.001
T1	23	3	7.67	2.55	23.08	<0.001
T2	4	3	1.33	0.32	5.50	0.500
T3	3	2	1.50	0.27	8.40	0.500
BG (mmol/L)	P_Mauchly_=0.002, Fgroup*time=80.91, Pgroup*time<0.001					
T0	5.43±0.57	5.51±0.50	0.08	-0.34	0.19	0.568
T1	9.00±1.27	5.46±0.51	3.53	3.06	4.01	<0.001
T2	6.54±1.24	5.06±0.44	1.48	1.02	1.94	<0.001
T3	5.05±0.54	5.15±0.53	−0.10	−0.36	0.163	0.452
INS (mIU/L)	P_Mauchly_ <0.001, Fgroup*time=185.73, Pgroup*time<0.001					
T0	11.01±3.89	11.23±3.72	−0.22	−2.09	1.66	0.819
T1	47.53±10.41	10.69±3.65	36.84	33.00	40.67	<0.001
T2	23.35±6.40	10.96±3.57	12.39	9.84	14.94	<0.001
T3	12.41±4.17	12.35±3.37	0.05	−1.82	1.91	0.959
HOMA-I R	P_Mauchly_ <0.001, Fgroup*time=310.86, Pgroup*time<0.001					
T0	2.68±1.04	2.76±0.96	−0.072	−0.57	0.42	0.770
T1	18.78±3.98	2.60±0.92	16.18	14.76	17.60	<0.001
T2	6.76±2.15	2.65±0.84	4.12	3.49	5.09	<0.001
T3	2.77±0.96	2.85±0.91	−0.09	−0.55	0.37	0.702
VAS of HT						
T0	1 (0,1)	1 (1,1)				0.005
T1	1 (0,1)	1 (1,1)				<0.001
T2	1 (1,1)	1 (1,2)				0.261
T3	1 (1,2)	2 (1,2)				0.012

MD, Mean difference; CI, Confidence interval; LL, Lower limit; UL, Upper limit; P_Mauchly_, P value for Mauchly’s test of sphericity; Fgroup × time, F-statistic for the Group × Time interaction; Pgroup × time, P-value for the Group × Time interaction; CSA, Cross-sectional area of the antrum of the stomach; GER, Gastric Emptying Rate; GET, Gastric Emptying Time; HRS, High-Risk Stomach; OR, Odds ratio.

Repeated-measures analysis of variance (RM-ANOVA) revealed a statistically significant group × time interaction effect for CSA (*F* = 258.11, *p* < 0.001). Mauchly’s test of sphericity showed a violation of the sphericity assumption (*p* < 0.001), thus the Greenhouse–Geisser correction was applied. Simple effect analyses were performed to compare between-group differences at each time point:

- At T0: CSA in the experimental group was significantly higher than that in the control group (*p* < 0.001), with a mean difference (MD) of 7.78 cm^2^ (95% CI, 7.40 to 8.11).
- At T1: CSA in the experimental group remained significantly higher than that in the control group (*p* < 0.001), MD = 2.60 cm^2^ (95% CI, 2.16 to 3.03).
- At T2 (primary observation time point): There was no statistically significant difference in CSA between the two groups (*p* = 0.137), MD = 0.47 cm^2^ (95% CI, −0.15 to 1.10).
- At T3: There was no statistically significant difference in CSA between the two groups (*p* = 0.940), MD = −0.13 cm^2^ (95% CI, −0.36 to 0.336).

### 3.3 Secondary outcomes

#### 3.3.1 Gastric volume (GV)

RM-ANOVA showed a significant group × time interaction effect for GV (*F* = 260.49, *p* < 0.001) (Table 2). Mauchly’s test of sphericity was violated (*p* < 0.001), and the Greenhouse–Geisser correction was applied. Simple effect analyses were conducted for between-group comparisons at each time point:

- At T0: GV in the experimental group was significantly higher than that in the control group (*p* < 0.001), MD = 113.24 mL (95% CI: 105.31 to 121.16).
- At T1: GV in the experimental group was significantly higher than that in the control group (*p* < 0.001), MD = 37.91 mL (95% CI: 28.72 to 47.10).
- At T2: There was no statistically significant difference in GV between the two groups (*p* = 0.278), MD = 6.76 mL (95% CI: −5.58 to 19.09).
- At T3: There was no statistically significant difference in GV between the two groups (*p* = 0.973), MD = −0.16 mL (95% CI: −9.12 to 9.43).

At the primary observation time points T2 and T3, gastric volume in all participants did not exceed the recognized safety threshold of 1.5 mL/kg.

#### 3.3.2 Incidence of high-risk stomach (HRS)

The incidence of high-risk stomach was analyzed using Fisher’s exact test, and the results were as follows:

- At T0: The incidence of high-risk stomach in the experimental group was significantly higher than that in the control group (*p* < 0.001), with an odds ratio (OR) of 8.25 (95% CI: 3.29 to 20.67).
- At T1: The incidence of high-risk stomach in the experimental group was significantly higher than that in the control group (*p* < 0.001), OR = 7.67 (95% CI: 2.55 to 23.08).
- At T2 and T3: There were no statistically significant differences in the incidence of high-risk stomach between the two groups (both *p* = 0.500), with OR values of 1.33 (95% CI: 0.32 to 5.50) and 1.50 (95% CI: 0.27 to 8.40), respectively.

#### 3.3.3 Blood glucose (BG)

RM-ANOVA revealed a significant group × time interaction effect for BG (*F* = 80.91, *p* < 0.001). Mauchly’s test of sphericity was violated (*p* = 0.002), and the Greenhouse–Geisser correction was applied. Simple effect analyses were performed for between-group comparisons at each time point:

- At T0, T2 and T3: There were no statistically significant differences in BG between the experimental group and the control group (all *p* > 0.05);
- At T1: BG in the experimental group was significantly higher than that in the control group (*p* < 0.001), MD = 3.53 mmol/L (95% CI: 3.06 to 4.01).

#### 3.3.4 Insulin (INS)

RM-ANOVA showed a significant group × time interaction effect for INS (*F* = 185.73, *p* < 0.001). Mauchly’s test of sphericity was violated (*p* < 0.001), and the Greenhouse–Geisser correction was applied. Simple effect analyses were conducted for between-group comparisons at each time point:

- At T0 and T3: There were no statistically significant differences in INS between the two groups (all *p* > 0.05).
- At T1 and T2: INS in the experimental group was significantly higher than that in the control group (both *p* < 0.001), with MD values of 36.84 mIU/L (95% CI: 33.00 to 40.67) and 12.39 mIU/L (95% CI: 9.84 to 14.94), respectively.

#### 3.3.5 Homeostasis model assessment of insulin resistance (HOMA-IR)

RM-ANOVA revealed a significant group × time interaction effect for HOMA-IR (*F* = 310.86, *p* < 0.001). Mauchly’s test of sphericity was violated (*p* < 0.001), and the Greenhouse–Geisser correction was applied. Simple effect analyses were performed for between-group comparisons at each time point:

- At T0 and T3: There were no statistically significant differences in HOMA-IR between the two groups (all *p* > 0.05), with MD values of −0.072 (95% CI: −0.57 to 0.42) and −0.09 (95% CI: −0.55 to 0.37), respectively.
- At T1 and T2: HOMA-IR in the experimental group was significantly higher than that in the control group (both *p* < 0.001), with MD values of 16.18 (95% CI: 14.76 to 17.60) and 4.12 (95% CI: 3.49 to 5.09), respectively.

#### 3.3.6 Visual analogue scale scores for hunger and thirst (HT-VAS)

HT-VAS data were non-normally distributed and presented as median (interquartile range, IQR). Between-group comparisons were performed using the Mann–Whitney U test:

- At T0 and T1: There were statistically significant differences between the two groups (*p* = 0.005 and *p* < 0.001, respectively).
- At T2: There was no statistically significant difference between the two groups (*p* = 0.261).
- At T3: The severity of hunger and thirst in the experimental group was significantly lower than that in the control group (*p* = 0.012).

## 4 Discussion

This was a single-center, randomized, controlled, non-inferiority clinical trial that used point-of-care gastric ultrasound to evaluate gastric content status and safety following oral administration of 12.5% carbohydrate beverage 2 h preoperatively in healthy volunteers after prolonged fasting. The primary outcome showed that antral cross-sectional area (CSA) in the right lateral decubitus position at 2 h (T2) and 3 h (T3) after beverage ingestion in the experimental group was non-inferior to that in the control group with continuous fasting. Moreover, gastric volume in all participants did not exceed the safety threshold of 1.5 mL/kg at the primary observation time points. Meanwhile, preoperative oral carbohydrate significantly alleviated subjective discomforts such as hunger and thirst, without causing a sustained increase in insulin resistance. These findings provide objective evidence for the safety and clinical feasibility of oral carbohydrate administration 2 h before surgery.

Pulmonary aspiration is a highly dangerous complication in the peri-anesthetic period, and strict preoperative fasting has long been adopted as the core preventive strategy. However, numerous studies have confirmed that excessively prolonged fasting leads to thirst, hunger, anxiety, exaggerated stress responses, and insulin resistance, thereby impairing postoperative recovery (4). To balance safety and patient comfort, both the American Society of Anesthesiologists (ASA) and relevant Chinese guidelines recommend clear fluid or carbohydrate intake up to 2 h preoperatively, which has become an important component of Enhanced Recovery After Surgery (ERAS) (6, 7). Nevertheless, unduly prolonged preoperative fasting remains prevalent in clinical practice due to traditional beliefs, with the major concern being that retained gastric contents may increase the risk of aspiration (8, 10).

This study selected antral CSA in the right lateral decubitus position as the primary outcome. Previous studies and our team’s earlier review have verified that gastric volume (GV) is calculated by substituting CSA into regression formulas, but different formulas yield large discrepancies and poor stability in the low-volume range, and may even produce physiologically meaningless negative values (8). In contrast, antral CSA is a directly measured continuous variable that is objective, stable, and highly reproducible, and is internationally recognized as a key indicator for assessing gastric contents (15). The non-inferiority results of this study demonstrated no significant difference in CSA between the experimental and control groups at T2 and T3, with all values within the safe range, directly confirming that oral carbohydrate administration 2 h preoperatively does not delay gastric emptying. The non-inferiority margin (NIM) of CSA in this study (2.0 cm^2^) is derived from the difference between Perlas grade 0 (CSA = 3.6 ± 1.0 cm^2^) and Perlas grade 1 (CSA = 5.6 ± 1.4 cm^2^) reported in the study by Perlas et al. (15). They clearly stated that grade 1 corresponds to a safe low volume of gastric contents (16 ± 36 mL; 0.2–0.5 mL/kg), while grade 0 corresponds to a completely empty stomach. Based on this, we set the NIM at 2.0 cm^2^ to reflect the clinically acceptable difference between these two states. This NIM setting is also supported by similar previous studies: Eun-Ah Cho et al. set the CSA non-inferiority margin at 2.8 cm^2^, whereas the NIM of 2.0 cm^2^ in this study is stricter (16). Therefore, the selection of NIM = 2.0 cm^2^ is based on clear clinical evidence, fully considers the clinical significance of CSA differences, and adopts the complete difference between Perlas grade 0 and grade 1, which aligns with the purpose of this study to evaluate the safety of gastric emptying between preoperative carbohydrate drinks and prolonged fasting.

In the present study, gastric volume in all participants was below 1.5 mL/kg at T2 and T3, further supporting the safety of the intervention. Although controversy remains regarding the critical threshold for high-risk gastric volume, thresholds such as 0.4 mL/kg and 0.8 mL/kg are mostly derived from animal studies and cannot be directly extrapolated to clinical settings (17–19), whereas 1.5 mL/kg has been widely accepted as a clinically applicable safety threshold for gastric volume (11). Our findings suggest that in healthy individuals, gastric contents return to nearly fasting levels 2 h after carbohydrate ingestion following prolonged fasting. In addition, subjects in group E were designed to receive individualized intake of the carbohydrate beverage with a maximum allowed volume of 400 mL. The actual intake was 221 ± 51 mL (range, 150–350 mL), and no participant consumed the full 400 mL. Therefore, the safety conclusions of this study are strictly applicable to the intake range of 150–350 mL, rather than the full up-to-400 mL range stated in the Methods section. This pattern reflects the natural voluntary drinking behavior of healthy volunteers, and extrapolation of the present findings to intake volumes exceeding 350 mL should be made with caution.

For metabolic parameters, blood glucose, insulin, and HOMA-IR were significantly elevated in the experimental group at T1 and T2, representing normal physiological responses to carbohydrate intake. By T3, there was no statistically significant difference in HOMA-IR between the two groups, and values in the experimental group were slightly lower than those in the control group. These results indicate that preoperative oral carbohydrate does not induce sustained insulin resistance, but may instead alleviate metabolic stress caused by prolonged fasting. Perioperative insulin resistance is mainly attributed to prolonged fasting (20) and is closely associated with postoperative infection, cardiovascular complications, and delayed recovery. Preoperative carbohydrate administration attenuates stress and maintains metabolic homeostasis, which is consistent with the ERAS concept (21).

Regarding subjective symptoms, VAS scores for hunger and thirst in the experimental group were significantly better than those in the control group, with a more pronounced difference at T3. This finding is consistent with multiple studies showing that shortening the preoperative fluid fasting period significantly improves patient comfort, reduces anxiety and irritability, and enhances the overall medical experience (22–24).

This study has several limitations. First, it enrolled ASA Class I healthy volunteers rather than surgical patients. Surgical stress, anesthetic agents, obesity, diabetes, and other conditions may slow gastric emptying and exacerbate insulin resistance; therefore, the present conclusions cannot be directly extrapolated to surgical populations (11, 25). Second, gastric fluid pH was not measured. Gastric pH is a key determinant of the severity of lung injury following aspiration; however, for ethical reasons, invasive gastric fluid aspiration was not performed in healthy volunteers, and this indicator may be included in future clinical studies (10, 22). Third, some controversy remains regarding the criteria for defining a high-risk stomach, and no universal threshold applicable to all populations has been established. The multi-index composite judgment used in this study may improve sensitivity but may reduce specificity to a certain extent (11). Fourth, all ultrasound measurements were performed by a single operator, and the CSA results were entirely dependent on that operator’s technique and judgment; therefore, inter-rater reliability could not be assessed.

In conclusion, in healthy volunteers, oral carbohydrate beverage 2 h preoperatively after prolonged fasting results in non-inferior antral CSA compared with the conventional fasting regimen at 2 h after ingestion. It also significantly relieves discomforts such as hunger and thirst, without aggravating insulin resistance.

## Data Availability

The original contributions presented in the study are included in the article/supplementary material, further inquiries can be directed to the corresponding author.
